# Integrated nanophotonic hubs based on ZnO-Tb(OH)_3_/SiO_2 _nanocomposites

**DOI:** 10.1186/1556-276X-6-503

**Published:** 2011-08-22

**Authors:** Hsia Yu Lin, Chung Liang Cheng, Yu Shen Lin, Yann Hung, Chung Yuan Mou, Yang Fang Chen

**Affiliations:** 1 Department of Physics, National Taiwan University, No. 1, Sec. 4, Roosevelt Rd, Taipei 106, Taiwan, Republic of China; 2 Department of Chemistry, National Taiwan University, No. 1, Sec. 4, Roosevelt Rd, Taipei 106, Taiwan, Republic of China

**Keywords:** ZnO, Tb(OH)_3_/SiO_2_, nanocomposite, lasing

## Abstract

Optical integration is essential for practical application, but it remains unexplored for nanoscale devices. A newly designed nanocomposite based on ZnO semiconductor nanowires and Tb(OH)_3_/SiO_2 _core/shell nanospheres has been synthesized and studied. The unique sea urchin-type morphology, bright and sharply visible emission bands of lanthanide, and large aspect ratio of ZnO crystalline nanotips make this novel composite an excellent signal receiver, waveguide, and emitter. The multifunctional composite of ZnO nanotips and Tb(OH)_3_/SiO_2 _nanoparticles therefore can serve as an integrated nanophotonics hub. Moreover, the composite of ZnO nanotips deposited on a Tb(OH)_3_/SiO_2 _photonic crystal can act as a directional light fountain, in which the confined radiation from Tb ions inside the photonic crystal can be well guided and escape through the ZnO nanotips. Therefore, the output emission arising from Tb ions is truly directional, and its intensity can be greatly enhanced. With highly enhanced lasing emissions in ZnO-Tb(OH)_3_/SiO_2 _as well as SnO_2_-Tb(OH)_3_/SiO_2 _nanocomposites, we demonstrate that our approach is extremely beneficial for the creation of low threshold and high-power nanolaser.

## Background

Semiconductive photonic nanostructures have attracted increasing attention for its many possible applications, such as laser, solar cell, biosensor, and photoelectric conversion [1-4]. Among all the semiconductor materials, zinc oxide is of great interest for photonic applications due to its wide bandgap (3.37 eV) and efficient emission [5]. The optoelectronic properties of zinc oxide depend critically on its defect structure and rich morphologies. ZnO nanostructures have been made into diverse morphologies, such as nanoparticles, nanorods, nanowires, nanobelts, and nanotubes [6-9]. Of these, ZnO nanorods have received the greatest attention and have shown to be a good laser emitter, an electron emitter, and a photoelectric converter. Their excellent optical behaviors are due to the fact that ZnO cannot only be a good gain medium but also can present good confinements for both photons and electrons. Numerical calculations have concluded that ZnO nanorods provide high lateral photonic confinement and are excellent waveguides [10]. Light intensity losses occur only at the end faces, and this makes longer nanorods higher *Q *resonators. In addition, nanostructures like ZnO nanorods coupled with photonic nanomaterials can lead to newer applications.

When another optical nanostructure is coupled with ZnO, the integrated optical phenomenon can be demonstrated. We would like to study the coupling of ZnO and the strong luminescent nanomaterials of lanthanide hydroxide. Due to the unique electronic, optical, and magnetic properties arising from the 4*f *electrons, lanthanide hydroxides are very attractive in various applications, including catalysts, laser materials, biolabels, and magnetic resonance imaging [11]. Previously, lanthanide-doped nanoparticles have been fabricated mainly by ion implantation [12], sol-gel method [13], and sonochemical synthesis [14]. Unfortunately, the obtained size is often not uniform. Recently, we reported a one-pot synthesis of monodispersed core/shell Tb(OH)_3_/SiO_2 _colloids [15]. The Tb(OH)_3_/SiO_2 _colloidal particles self-assembled into a 3-D photonic crystals (PCs), giving a pronounced optical gap depending on the particle size. Many efforts have been made on applications of PCs, such as the resonators, sensors, and reflectors [16-18]. To expand more applications of Tb(OH)_3_/SiO_2 _with other materials and nanostructures, semiconductor nanowires were chosen because they can be used as waveguides when attached to other luminescent materials [19]. Based on the monodispersed Tb(OH)_3_/SiO_2 _core/shell nanoparticles, we report a novel composite with ZnO nanotip on Tb(OH)_3_/SiO_2 _core/shell nanoparticle (ZnO-Tb(OH)_3_/SiO_2_), which can be used to manipulate the emissions from inside the PCs. Due to the confinement effect of PC, emissions can escape only from the nanotips of ZnO. We found that the light output can be greatly enhanced by two orders of magnitude. To optimize this effect, SnO_2 _nanowires were selected to show the enhanced lasing emission of Tb(OH)_3_/SiO_2 _PCs by growing them on Tb(OH)_3_/SiO_2 _PCs of 130 nm which can perform better lasing action at 380 nm. Therefore, these novel composites act like directional light fountains, i.e., the light confined underneath the surface of the photonic crystal can be extracted only through the specially designed semiconductor nanotips. We show that this unique property is very useful to create low threshold and high-power nanolasers.

## Methods

Nanoparticles of Tb(OH)_3 _were encapsulated inside silica as core/shell structures with an outer diameter of 250 nm by a one-pot synthesis method reported in our previous paper [15]. The monodispersed nanoparticles were self-assembled on glass or Si (100) substrate by a slow evaporation method, resulting in self-organized packing as photonic crystals. After coating with gold nanoparticles (20 mA, 20 s), Tb(OH)_3_/SiO_2 _nanoparticles were used as templates in a vapor-liquid-solid process to grow ZnO nanowires on the nanospheres. The mixed C/Zn powders were placed in an alumina boat, which was loaded in the center of a tube furnace. The gold-coated lanthanide nanosphere substrate was placed in the same boat but apart from the mixed powders for about 3 cm. Argon was then introduced into the system with a flow rate of 200 sccm as the carrier gas. Afterwards, the tube was heated to 980°C at a rate of 40°C/min. The reaction lasted about 60 min. After the furnace cooled down, white color products formed on the surface of the lanthanide nanosphere substrate. For SnO_2 _nanowire growth, the C/Zn powders were replaced with C/Sn powders then follow the above steps. Cathodoluminescence (CL) experiments were performed at room temperature with a scanning electron microscopy (JSM 6500, JEOL Ltd., Tokyo, Japan). Excitation spectra were gathered by a PMT detector with a CL system (Gatan instrument, MonoCL3, Gatan, Inc., Pleasanton, CA, USA).

## Results and discussion

### Fabrication of ZnO-Tb(OH)_3_/SiO_2 _composites

**Figure 1:**
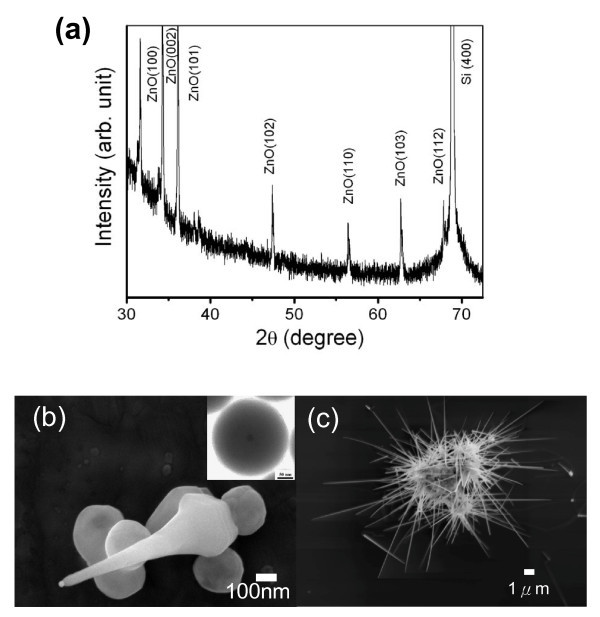
**Fabrication of ZnO-Tb(OH)_3_/SiO_2 _composites**. **(a) **XRD pattern and **(b) **SEM image of an initially grown ZnO nanotip on a Tb(OH)_3_/SiO_2 _nanosphere. Inset is the TEM image of a single Tb(OH)_3_/SiO_2 _nanoparticle of 250 nm with the scale bar of 50 nm. **(c) **SEM image of ZnO nanotip composites, sea urchin-type.

When the ZnO nanotips are grown on Tb(OH)_3_/SiO_2 _nanospheres, they possess a wurtzite structure with a longer length of 15 μm (Figure 1a). In contrast, ZnO rods grown on sapphire under the same growth conditions are generally less than 5 μm long [20,21]. As ZnO nanotips were grown on Tb(OH)_3_/SiO_2 _nanospheres, it gradually transforms from a hexagonal to a conical shape (Figure 1b). The conical tip shape and the high aspect ratio (approximately 150) of nanotips are especially beneficial for field emission application. Generally, more than one ZnO nanotip can be grown on each nanosphere. For the aggregation of Tb(OH)_3_/SiO_2 _nanospheres, the composite of the ZnO nanotips and nanospheres appears like a sea urchin as shown in Figure 1c.

**Figure 2:**
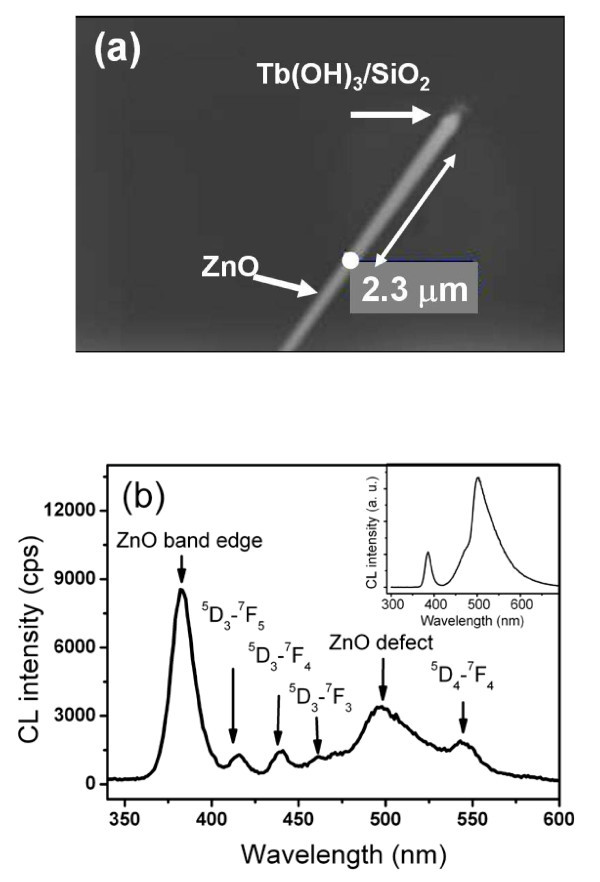
**ZnO nanotip adhering to a Tb(OH)_3_/SiO_2_**. **(a) **An electron beam was focused at a point (white dot) on the ZnO nanowire, which was grown on a Tb(OH)**_3_**/SiO**_2 _**nanosphere with an acceleration voltage of 10 keV. **(b) **Collected CL spectrum of (a). The inset is the CL spectrum of pure ZnO nanowires.

When a ZnO nanotip adheres to a Tb(OH)_3_/SiO_2 _nanosphere (Figure 2a), one can collect the emission of excited Tb(OH)_3_/SiO_2 _nanoparticles at the end of ZnO and vice versa. Due to the multiple transition bands of Tb^3+ ^(^5^D_3_-^7^F_6_, 381 nm; ^5^D_3_-^7^F_5_, 416 nm; ^5^D_3_-^7^F_4_, 439 nm; ^5^D_3_-^7^F_3_, 460 nm; ^5^D_4_-^7^F_6_, 491 nm; and ^5^D_4_-^7^F_5_, 546 nm; ^5^D_4_-^7^F_4_, 591 nm) [22] and band edge (380 nm) and defect emissions (500 nm) of ZnO, the complex emissions of ZnO-Tb(OH)_3_/SiO_2 _are illustrated with single ZnO nanotip-Tb(OH)_3_/SiO_2 _and urchin-like composites. As shown in Figure 2a, when a ZnO nanotip was excited by an electron beam at 2.3 μm apart from the center of Tb ion, the emissions of ZnO at 380 and 500 nm were propagated through the rod and then excited Tb giving rise to the CL spectrum emissions at 414, 438, 460, and 546 nm (Figure 2b). During the secondary excitation process, the Tb ion acts as a signal receiver and an emitter. Furthermore, as a result of the resonance $\left(\frac{\lambda }{n\cdot D}=N\right)$ between the propagating emission of ZnO at 380 nm (*λ*) and the SiO_2 _cavity (*n *= 1.5) with a diameter of 250 nm (*D*), the emission wavelength of ZnO at 380 nm in Tb(OH)_3_/SiO_2 _nanoparticles become 253 nm, which coincides with the cavity length. Thus, light can resonate inside the nanoparticles, and the detected emission with a wavelength of 380 nm outside the nanoparticle is highly enhanced.

**Figure 3:**
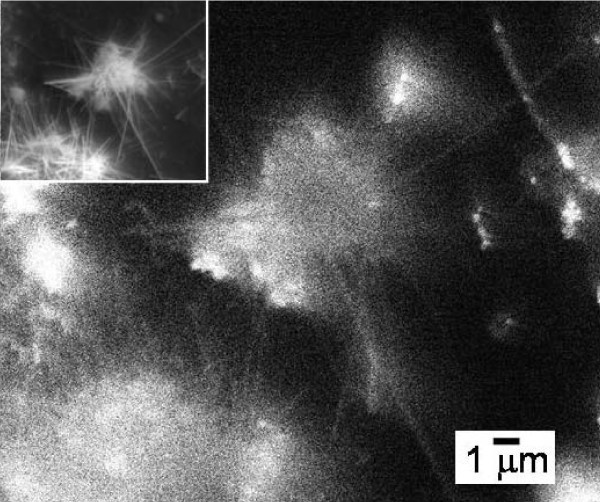
**Demonstration of the waveguide behavior of ZnO nanowires**. CL image of sea urchin-like ZnO-Tb(OH)_3_/SiO_2 _nanocomposites taken at 300 nm with an electron acceleration voltage of 15 keV. The inset is the corresponding SEM image.

For an urchin-like ZnO-Tb(OH)_3_/SiO_2 _composite, several ZnO nanotips diverge from the center as shown in the inset of Figure 3. Because several emissions of Tb overlap with the luminescence of ZnO nanotips, the defect emission at 300 nm which originated only from the defect state of silica was chosen to present the optical propagation properties. The lifetime of SiO_2 _can be extended to several seconds, depending on the calcination process, thus the emission of SiO_2 _can be detected by CL mapping [22]. For stand-alone ZnO nanowire, there is no emission at 300 nm, thus the detected emission at 300 nm is certainly propagated from Tb(OH)_3_/SiO_2_. As the Tb(OH)_3_/SiO_2 _sphere was excited, the emission can be propagated and detected along ZnO nanotips as shown in a monochromatic CL image taken at 300 nm (Figure 3). When excited, the emissions of Tb ion also can be dispatched from the pivot to the tips of ZnO. This behavior therefore proved that ZnO-Tb(OH)_3_/SiO_2 _can act as a light distributor/emitter, which enables the signal coming from the center to be distributed into the surrounding ZnO tips. In addition, it acts as an optical receiver, which is able to collect the light injected at the end of ZnO nanotips. As demonstrated above, these ZnO-Tb(OH)_3_/SiO_2 _nanocomposites can function as a waveguide, a receiver, an emitter, as well as a distributor. Therefore, the nanocomposite can serve as a multifunctional integrated nanophotonic hub, which serves as an efficient control for injected light.

### Photonic bandgap and CL spectra of Tb(OH)_3_/SiO_2 _PCs

**Figure 4:**
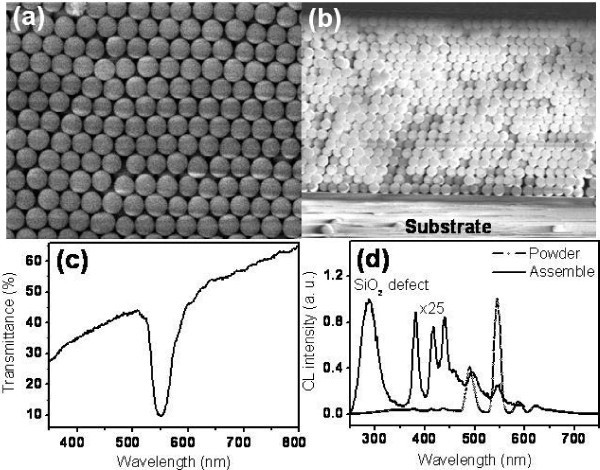
**Optical response of the assembled Tb(OH)_3_/SiO_2 _nanospheres and the resulting luminescence change**. **(a) **Top view and **(b) **lateral SEM images of assembled Tb(OH)_3_/SiO_2 _nanospheres. **(c) **Transmittance spectrum of (a). **(d) **CL spectrum of randomly dispersed Tb(OH)_3_/SiO_2 _(dotted line) and CL spectrum of assembled Tb(OH)_3_/SiO_2 _PCs (solid line), both were taken at an electron acceleration voltage of 15 kV.

**Figure 5:**
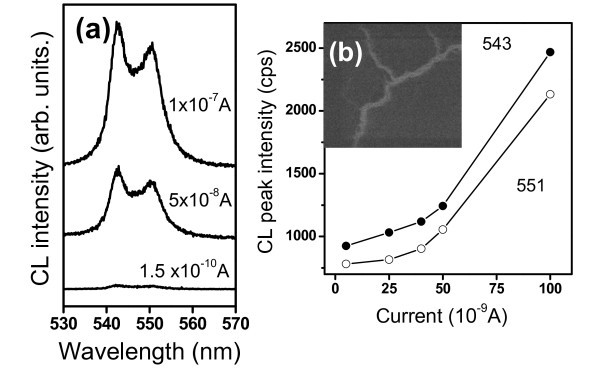
**CL properties showing the emissions of Tb(OH)_3_/SiO_2 _PCs under different excitation currents**. **(a) **Stimulated CL emission spectra of Tb(OH)_3_/SiO_2 _PCs with a diameter of 250 nm taken at an electron acceleration voltage of 15 kV. **(b) **Emission intensity at 543 and 551 nm vs the excitation current. The inset is the CL image taken at full wavelength with an acceleration voltage of 5 kV.

The top and lateral SEM images show assembled Tb(OH)_3_/SiO_2 _PCs (250 nm diameter) of about 20 to 25 layers (as shown in Figure 4a, b). As the nanospheres self-assembled into a face-centered cubic structure, it formed a stop band along the Γ to L direction which gives a sharp drop in the transmittance spectrum at around 550 nm (Figure 4c). For a single Tb(OH)_3_/SiO_2 _nanoparticle, the CL spectrum exhibits several bright emission bands of transitions from D levels to F levels at room temperature, ranging from 350 to 650 nm (Figure 4d, dotted) [15]. The defect emission of silica at 300 nm was extremely weak in comparison to the luminescence of Tb ion. As the nanoparticles self-assembled, the stop band effect led to the modification of the emissions for the Tb ion embedded in the PCs so that most emission bands were quenched as shown by the solid curve in Figure 4d. Note that the solid curve has been enlarged by 25 times compared with that of the dotted line. As the luminescence of the Tb ions was suppressed, the defect emission of silica at 300 nm became more pronounced. For Tb(OH)_3_/SiO_2 _PCs, little emission could be detected in the well-packed region due to the optical trap of the stop band. However, a CL image shows that the confined emissions of the Tb ions can escape from the crack region and be detected along the crack defects (inset of Figure 5b).

As the electron beam with a spot size of 1 μm to approximately 2 μm in diameter was focused on the Tb(OH)_3_/SiO_2 _PCs surface, CL spectra showed broadened bands of intra-4*f *transitions under a small excitation current. As the current reached 5 × 10^-8 ^A, two sharp peaks of the CL spectrum appeared at 543 and 551 nm (Figure 5a), possibly due to the Stark effect of stimulated emission [23]. With increasing current, the two peaks are more resolved, and the corresponding intensity increases nonlinearly as shown in Figure 5b. The nonlinear relationship between CL intensity and excitation current revealed a threshold current of 5 × 10^-8 ^A, indicating a stimulated emission behavior. PCs have been used as a lasing cavity to stimulate the confined emission inside [18]. The low threshold may arise from the release of optical resonance between emissions near the stop band and the cavity of PCs.

### Lasing action of rice paddy-like ZnO-Tb(OH)_3_/SiO_2 _PCs nanocomposites

**Figure 6:**
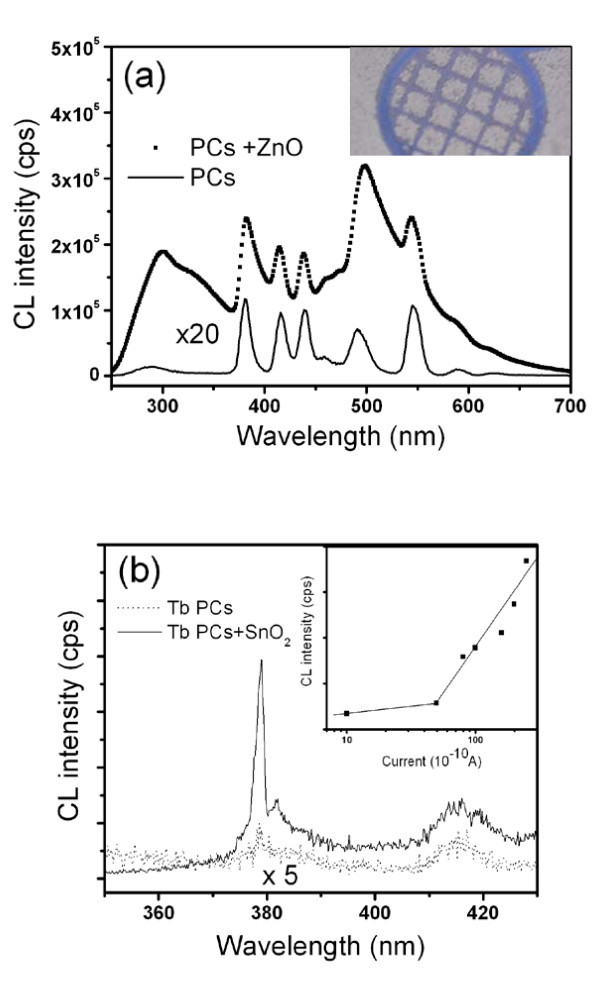
**Enhancement on the stimulated emission of Tb(OH)_3_/SiO_2 _PCs by using ZnO nanowires**. CL spectra of only Tb(OH)_3_/SiO_2 _PCs (solid line) and ZnO nanotips-Tb(OH)_3_/SiO_2 _PCs (dotted line) taken at an electron acceleration voltage of 20 keV. Inset is the optical image of rice paddy-like ZnO-Tb(OH)_3_/SiO_2 _PCs nanocomposites. White regions denote the Tb(OH)_3_/SiO_2 _PCs with ZnO nanowires. (b) Emission spectra of Tb(OH)_3_/SiO_2 _PCs with and without SnO_2 _nanowires under the excitation current of 8 × 10^-9 ^A. Inset is the emission intensity of Tb(OH)_3_/SiO_2 _with SnO_2 _nanowires vs excitation current.

After ZnO nanotips were grown on patterned Tb(OH)_3_/SiO_2 _PCs, they formed as rice paddy-like ZnO-Tb(OH)_3_/SiO_2 _PCs nanocomposites (inset of Figure 6). At least 40 times CL intensity was obtained in comparison to that of Tb(OH)_3_/SiO_2 _PCs (Figure 6a). Giant optical enhancement was found in ZnO-Tb(OH)_3_/SiO_2 _PCs that the previously diminished emission of PCs was enhanced through the ZnO nanotips. This enhancement demonstrated that in addition to defects, the confined emission of Tb ions can be released by propagating along ZnO nanotips. Under the circumstances, the ZnO nanotips act like a directional light fountain, in which the confined radiation inside PCs can escape from ZnO nanotips. In retrospection of the development of lasing mode or optical resonance, attention has been mostly focused on materials or structures. However, the purpose of the waveguide adapted lasing cavity is to reduce the loss of propagated emission in omnidirection and collimate light in a specific direction. As a result, the output emission intensity can be greatly enhanced. Similarly, once the lasing mode of the PCs is excited, the emission can be efficiently guided. To explore this intriguing possibility, Tb(OH)_3_/SiO_2 _PCs with a diameter of 130 nm were used to demonstrate the amplified laser action. Figure 6b shows emission spectra of Tb(OH)_3_/SiO_2 _PCs with and without SnO_2 _nanowires under the same excitation current of 8 × 10^-9 ^A. After the SnO_2 _nanowires were grown on Tb(OH)_3_/SiO_2 _PCs, the peak intensity near 380 nm has risen up to 20 times, and the full width at half maximum (FWHM) is about 2 nm. The lasing peak at 380 nm away from the stop band at 330 nm is attributed to the band edge lasing operation [24]. The inset of Figure 6b shows the dependence of emission intensity on exciting energy for the Tb(OH)_3_/SiO_2 _PCs with SnO_2 _nanowires. Without the aid of SnO_2 _nanowires, the threshold for laser emission of Tb(OH)_3_/SiO_2 _PCs is evaluated at 4 × 10^-8 ^A based on the result shown in Figure 5b. However, after SnO_2 _nanowires were grown on Tb(OH)_3_/SiO_2 _PCs, the threshold is reduced to 5 × 10^-9 ^A.

## Conclusions

With the designed ZnO-Tb(OH)_3_/SiO_2 _nanocomposite, a multifunctional integrated nanophotonic hub has been created. We have shown that growing ultra tapered ZnO nanotips on Tb(OH)_3_/SiO_2 _PCs can yield good control of emission out of PCs, in which the radiation confined underneath the PC surface can be well guided by the attached ZnO nanotips and escape from a designed direction. Similarly, SnO_2 _nanowires act as a directional light fountain, which may be very useful for the creation of ultra low threshold and high-power nanolaser. In view of the novel properties discovered here, the semiconductor Tb(OH)_3_/SiO_2 _composites pave a new way for the realization of applications of nanophotonics.

## Abbreviations

CL: cathodoluminescence; PCs: photonic crystals; SEM: scanning electron microscopy; VLS: vapor-liquid-solid.

## Competing interests

The authors declare that they have no competing interests.

## Authors' contributions

HYL designed the structure and did the SEM, CL, and PL measurements. CLC carried out the ZnO growth and XRD characteristics. YSL and YH synthesized Tb(OH)_3 _nanoparticles and assembled them. CYM and YFC participated in the structure design and application. All authors read and approved the final manuscript.
